# Tumour Microenvironment and Immune Evasion in EGFR Addicted NSCLC: Hurdles and Possibilities

**DOI:** 10.3390/cancers11101419

**Published:** 2019-09-24

**Authors:** Antonio Santaniello, Fabiana Napolitano, Alberto Servetto, Pietro De Placido, Nicola Silvestris, Cataldo Bianco, Luigi Formisano, Roberto Bianco

**Affiliations:** 1 Department of Clinical Medicine and Surgery, University of Naples “Federico II”, 80131 Naples, Italy; antonio.santaniello3@unina.it (A.S.); fabiana.napolitano@unina.it (F.N.); albeservetto@gmail.com (A.S.); pietrodep91@gmail.com (P.D.P.); 2 Medical Oncology Unit, IRCCS Istituto Tumori “Giovanni Paolo II” of Bari, 70124 Bari, Italy; silvestrisnicola@gmail.com; 3 Department of Biomedical Science and Human Oncology, University of Bari “Aldo Moro”, 70124 Bari, Italy; 4 Department of Experimental and Clinical Medicine, University of Catanzaro “Magna Graecia”, 88100 Catanzaro, Italy; bianco@unicz.it

**Keywords:** immune checkpoint inhibitors, ICI, EGFR mutation, NSCLC, angiogenesis, TKI, tumour microenvironment, TME, tumour mutational burden, TMB

## Abstract

In the last few years, the treatment strategy in Non-Small Cell Lung Cancer (NSCLC) patients has been heavily modified by the introduction of the immune-checkpoint inhibitors. Anti-programmed cell death 1/programmed cell death ligand 1 (PD-1/PD-L1) therapy has improved both progression-free and the overall survival in almost all subgroups of patients, with or without PDL1 expression, with different degrees of responses. However, there are patients that are not benefitting from this treatment. A defined group of immune-checkpoint inhibitors non-responder tumours carry EGFR (epidermal growth factor receptor) mutations: nowadays, anti-PD-1/PD-L1 clinical trials often do not involve this type of patient and the use of immune-checkpoint inhibitors are under evaluation in this setting. Our review aims to elucidate the mechanisms underlying this resistance: we focused on evaluating the role of the tumour microenvironment, including infiltrating cells, cytokines, secreted factors, and angiogenesis, and its interaction with the tumour tissue. Finally, we analysed the possible role of immunotherapy in EGFR mutated tumours.

## 1. Introduction

Lung cancer is the second most common cancer worldwide and the leading cause of cancer death for men and women. Non-Small Cell Lung Cancer (NSCLC), accounting for 85% of all lung cancer diagnoses, is the most common type [1,2]. Patients are often diagnosed in advanced stages and the prognosis is still very poor, with a five year survival of less than 10% [3]. 

In the adenocarcinoma subtype, activating mutation in the epidermal growth factor receptor (EGFR) gene occurs in approximately 50% of Asian patients and 10–15% of Caucasians [4]. EGFR is a member of the ErbB/HER (human epidermal growth factor) kinases family, and is involved in cancer cell proliferation, survival, and growth [5]. In this subset of tumours, over the last 10 years, significant improvements in progression free survival (PFS) has been achieved through the definition of a tailored treatment against the so-called “EGFR-sensitizing mutations” and the development of first-, second-, and third-generation tyrosine kinases inhibitors (TKIs) [6,7,8,9,10,11,12,13]. The introduction of immune checkpoint inhibitors (ICIs) marked the start of a new era of treatment strategies, with the achievement of great results regarding overall survival (OS) as the first- or second-line of treatment in Non-Small Cell lung cancer, as well as in other tumour types [14]. 

However, despite several preclinical studies showing a consistent positive correlation between EGFR mutation and programmed cell death ligand 1 (PD-L1) expression, both in cell lines [15,16] and in patients’ tissue samples [17,18], EGFR mutated tumours consistently showed no response to anti-PD-1/anti-PD-L1 treatment across most of the clinical trials. Indeed, in the CheckMate 057 trial, where nivolumab was compared with docetaxel as a second line therapy in advanced NSCLC patients, the EGFR mutated group had a better prognosis when treated with chemotherapy [19]. Similar results were obtained in other trials in the same setting (second line treatment in advanced NSCLC), such as KEYNOTE-010, pembrolizumab versus docetaxel [20], and the POPLAR and OAK trials, atezolizumab versus docetaxel [21,22]. Moreover, when EGFR-mutated patients were treated with concomitant chemotherapy and radiotherapy followed by maintenance therapy with durvalumab, patients receiving immunotherapy did not derive a clear benefit [23]. In addition to their failure as monotherapy treatments, the combination of immunotherapy and TKIs did not result in a synergistic anti-tumour effect, neither in cell lines carrying EGFR mutations and PD-L1 upregulation [16] nor in patients [24,25,26,27]. 

Most of our knowledge about EGFR mutated patients treated with ICIs comes from subgroup analysis of larger clinical trials and the few dedicated prospective trials [27,28] did not demonstrate a clear clinical benefit.

In this review, we aim to further elucidate the putative mechanisms of resistance and unresponsiveness to immunotherapy treatment in patients with EGFR-mutated NSCLC.

## 2. PD-L1 Expression in EGFR Mutated Patients 

Programmed cell Death Ligand 1 (PD-L1) is broadly expressed on the surface of tumour and immune cells across tumour types and its expression can protect tumours from cytotoxic T cells activity. Indeed, PD-L1 interacting with PD-1, expressed on the T cell surface, abrogates the T cells’ cytotoxic effect [29,30]. 

In 2013, Akbay et al., [15] demonstrated that EGFR mutations in bronchial epithelial cells induced PD-L1 expression, which was further reduced by TKIs. These results were confirmed on 164 surgical resected NSCLC tumour samples by Azuma and colleagues, where EGFR mutation was an independent factor that was directly correlated to PD-L1 increased expression [31]. D’Incecco and colleagues [17] confirmed the Azuma preclinical data, demonstrating a significant correlation between PD-L1 expression and the adenocarcinoma subtype and EGFR mutations. Finally, Tang et al., [32], analysing 170 lung adenocarcinoma patients’ tissues through immunohystochemical analysis, demonstrated that PD-L1 overexpression is more likely to be correlated with EGFR mutation. In contrast to these results, two pooled analyses [33,34] demonstrated an inverse correlation. In their study, Soo and colleagues evaluated the data of 3969 patients’ tissue samples through immunohystochemical analysis from 18 studies and found that EGFR mutant NSCLCs were less likely to be PD-L1-positive compared to wild-type EGFR tumors (odds ratio (OR) 0.59, 95% C.I. 0.39–0.92, *p* < 0.02) [33]. Also, Dong et al., [34] found that EGFR wild-type NSCLC tumors were more likely to be PD-L1 positive then EGFR mutant tumors (OR 1.79, 95% C.I. 1.10–2.93; *p* = 0.02). Moreover, analyzing mRNA and protein levels of PD-L1 in the Cancer Genome Atlas (TCGA) and internal database (Guangdong Lung Cancer Institute; GLCI), lower PD-L1 mRNA and PD-L1 protein expression were detected in the EGFR mutated NSCLC samples compared with the wild-type tumor samples [34]. However, these contrasting results could be related to differences in PD-L1 expression evaluation (i.e., immunohistochemistry assay versus mRNA expression) and interpretative scores among the others. A clear answer about the relationship between EGFR mutation and PD-L1 expression is not available yet.

## 3. Tumour Microenvironment 

The controversial data regarding EGFR mutation and PD-L1 expression did not clarify the reason for ICI ineffectiveness. It has been associated with impairment in the immune system, expressed as an uninflamed tumour microenvironment (TME), with immunological tolerance, and weak immunogenicity [34]. 

In 2004, the concept of cancer immunoediting was introduced to explain the relationship between the tumour and its microenvironment [35]. This process evolves in three steps: firstly, the cancer cells are eliminated by the host immune system (elimination phase). Following this, there is a phase of equilibrium, in which the immune system is not able to completely erase the tumour, inducing, in addition, a selective pressure on the tumour clones. Finally, the cancer cells develop the ability to evade the immune surveillance and grow uncontrollably (evasion). This study highlighted the importance of the TME in tumour development and progression. The TME of EGFR mutated NSCLC, as in other tumours [36], includes several cells of the immune system: tumour-associated macrophage (TAM), T cell CD4+, CD8+, regulatory T cell (Treg FOXP3+), and mast cells [37]. The interaction between tumour cells and TME is dynamic. Wycoff and colleagues [38] demonstrated how TAMs, usually located at the edge of the tumours, are stimulated by colony stimulating factor 1 (CSF1) to produce epidermal growth factor (EGF), the EGFR natural ligand. This interaction stimulated the EGFR downstream pathway, promoting cell growth and survival. Moreover, it was demonstrated that PD-L1 expression was increased in EGFR mutated cell lines after EGF stimulation [16]. 

**Figure 1:**
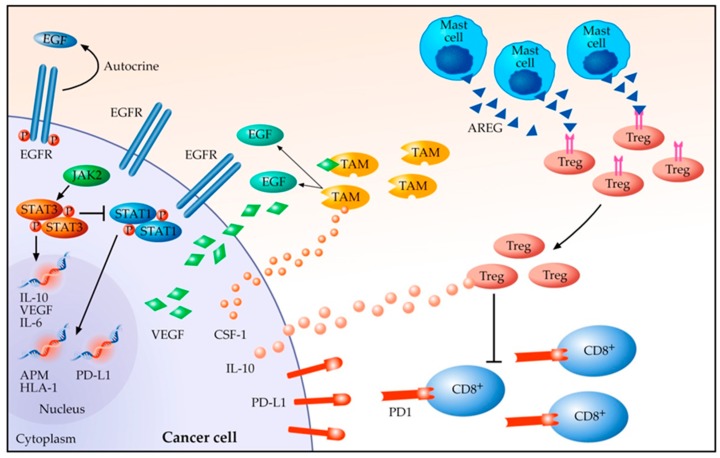
Tumour Microenvironment in EGFR mutated NSCLC. EGF, Epidermal Growth Factor; EGFR, Epidermal Growth Factor Receptor; STAT1/3, Signal Transducer and Activator of the Transcription1/3; JAK2, Janus Activated Kinase 2; IL, Interleukin, VEGF, Vascular Endothelial Growth Factor; APM, Antigen Presenting Machine; HLA-1, Human Leukocyte Antigen-1; PD-L1, Programmed cell Death Ligand 1; PD1, Programmed cell Death 1; CSF-1, Colony Stimulating Factor-1; TAM, Tumour Associated Macrophage; Treg, Regulatory T cell; CD8+, T cell CD8+; AREG, Amphiregulin; NSCLC, Non-Small Cell Lung Cancer.

PD-L1 was discovered as one of the primary immunosuppressive drivers in multiple types of cancers [39]. In addition to the PD-L1 expression, the immune evasion can be powered by the activity of mast cells and Treg cells, downregulating CD4+ and CD8+ T cells activity. Indeed, mast cells can release amphiregulin (AREG), which can interact with EGFR located on the Treg surface and, through a tonic signal, the MAPK pathway is constantly activated, thus resulting in Treg overactivation [40,41,42]. Tregs proliferation, together with myeloid derived suppressive cells (MDSC) and TAMs, can be induced by suppressive cytokines, while promoting unresponsiveness in CD8+ T cell effectors [43,44]. These cytokines can be induced by EGFR pathway stimulation. Indeed, its activation upregulates signal transducer and activator of the transcription 3 (STAT3) activity [45,46,47], which has a key role in the regulation of antigen presentation. STAT3 is able to inhibit STAT1 activity, which is finalized to the production of HLA1 (human leucocyte antigen 1) and of other components of the antigen presenting machine (APM) [48,49]. Moreover, STAT3 mediates the expression of vascular endothelial growth factor (VEGF), IL-6, and IL-10 and as demonstrated in many cancer types, these factors induce the inhibition of dendritic cells (DC) differentiation and maturation, and the result is T cell tolerance [50,51]. In Figure 1, the abovementioned interactions are shown.

## 4. Immunocheckpoint Inhibitors (ICIs) Treatment Failure 

The role of ICI in NSCLC patients has been investigated in several clinical trials, although in patients harbouring EGFR mutations, the expected endpoints were not met (Table 1). CheckMate 057 was a phase III trial investigating the role of the anti-PD-1 nivolumab in non-squamous advanced NSCLC patients, progressing during or after a platinum-based chemotherapy [19]. In this trial, patients with known EGFR mutation were allowed to receive an additional line of TKI therapy before enrolment. A subgroup analysis showed that the EGFR population that was involved in this study (14%) had better PFS and OS when treated with docetaxel rather than with nivolumab (HR PFS 1.45, 95% C.I. 0.90–2.37; HR OS 1.18 95% C.I. 0.69–2.00) [19]. These results were consistent with the subgroup analysis of EGFR mutated patients in the KEYNOTE-010 trial (HR OS 0.88, 95% C.I. 0.45–1.70 docetaxel versus pembrolizumab) [20] as well as in the POPLAR (phase II) and OAK (phase III) trials, comparing atezolizumab and docetaxel (HR OS 0.99, 95% C.I. 0.29–3.40 and HR OS 1.24, 95% C.I. 0.71–2.18, respectively) [21,22]. Consistently, a recent meta-analysis [52] evaluating only the abovementioned phase III trials revealed that ICIs were not superior to docetaxel for OS in the EGFR mutated patients subgroup (HR 1.05, 95% C.I. 0.70–1.55). In addition, the ATLANTIC trial evaluated the use of durvalumab as a third line of treatment (or more) in several cohorts of patients: the first cohort (number 1) included patients with EGFR mutations [53]. This study enrolled 97 patients harbouring EGFR mutations, of which 30 had a PD-L1 expression <25%. The objective response rates (ORR) evaluated per PD-L1 expression found a 12% response rate in the group with EGFR mutation and PD-L1 upregulation versus only 4% (1 patient with a partial response) in the group of patients with PD-L1 <25%. The OS in the EGFR mutated and PD-L1 <25% population was 9.9 months versus 13.3 months in the EGFR mutated and PD-L1 >25% group, with a similar PFS in both groups (1.9 months). However, the cohort size is too small to draw any conclusions and further studies are needed. 

Data from CheckMate 057, KEYNOTE-010, OAK, and POPLAR trials were recently evaluated together in a meta-analysis [54]. These studies involved 2753 patients, of which 272 (9.88%) had a known EGFR mutation: 44 patients were treated with nivolumab, 60 with pembrolizumab, 53 with atezolizumab, and 115 patients with docetaxel. The percentage of the EGFR mutated patients that were enrolled is representative of the EGFR mutated population of western countries (10–15% [4]). The meta-analysis showed a pooled HR OS of 1.12 (95% C.I. 0.85–1.38; heterogeneity *p* = 0.94) and a statistically significant treatment effect (*p* < 0.0001). Moreover, the SUCRA value (surface under the cumulative ranking curve) showed a higher rank (i.e., a higher area under the curve, meaning a better treatment performance) for docetaxel versus immunotherapy treatments: the SUCRA value for docetaxel was 60%, versus 48% for pembrolizumab, 46% for atezolizumab, and 45.6% for nivolumab. Although, this is a meta-analysis of few studies with a large variety of patient characteristics (number of previous lines, several EGFR mutations).

Despite the unsatisfactory experiences in the second line setting, ICIs were offered to TKI naïve EGFR mutated patients as a first line treatment: in a phase II trial single arm by Lisberg and colleagues [28], 1/11 patients showed an objective response when treated with Pembrolizumab. This result was obtained in PD-L1 positive patients, selected through an enriched design, where more than 70% of the patients had PD-L1 expression of more than 50%. However, further analysis on the tissue specimen derived from the single patient who had benefitted from pembrolizumab, revealed the absence of EGFR gene mutations.

These findings suggest that unresponsiveness to immunotherapy in the EGFR mutated NSCLC population could not be related to a previous treatment with TKI nor to PD-L1 low expression. The reduced efficacy of ICIs treatment in this cohort of patients should probably be searched in the TME characteristics. 

Indeed, several studies demonstrated a state of weak immunogenicity of the TME in EGFR mutated NSCLC patients. This non-inflamed TME may result from the absence of CD8+ T cell infiltrates, shrinking of PD-L1+/CD8+ TILs, and substantial reduction of the tumour mutational burden (TMB) [18,34]. The lack of effector cells may limit an anti-tumour immune response, even in the subset of patients with higher PD-L1 expression.

Recently, some interesting data correlated microbioma with the upregulation of PD-1 expression on lymphocytes and CD8+ T cell activation [55]. Moreover, the disturbance of lung microbioma composition (dysbiosis) found in heavy smokers is likely involved in the emergence of the inflammatory process (as in chronic obstructive pulmonary disease) and lung cancer [56]. In addition to this, there is evidence that even dysbiosis due to recurrent antibiotic treatments can be related to a higher risk of lung cancer [57]. The role of microbiota is currently under investigation as a possible mechanism of resistance to ICIs treatment. Indeed, it has been demonstrated that NSCLC patients treated with antibiotics within two months before or one month after the first administration of ICI had a significantly shorter PFS and OS when compared to untreated patients [56]. Similar results have been obtained by Derosa et al.,: NSCLC patients treated with antibiotics within 30 days from the beginning of ICI therapy had shorter PFS (1.9 months versus 3.8 months) and OS (7.9 months versus 24.6 months) [58]. These data conclude that the modulation of dybiosis and the microbioma may be an interesting strategy to improve clinical outcomes in patients treated with ICIs. However, further studies are needed to transpose these data to the subset of EGFR mutated patients.

Eventually, a combination of PD-1 or PD-L1 inhibitors and EGFR TKI were tested in several clinical trials, addressed to EGFR mutated TKI pre-treated or naïve patients (Table 2). Erlotinib was combined with nivolumab in a phase I trial, showing an ORR of 15% (3/20, including one complete response) [24]. In a phase Ib trial, erlotinib was combined with atezolizumab: the first results showed an ORR of 75% (95% C.I. 50.9–91.3), with a median duration of response (DOR) of 16.7 months (range, 4.2–26.0) [25]. The median PFS was 15.4 months (95% C.I. 8.4—not estimable [NE]), and the median OS was 32.7 months (95% C.I. 32.7—NE). Although there were no grade ≥4 adverse events, grade 3 treatment-related AEs (TRAEs) were reported in 43% of patients [25]. Indeed, toxicities were a major concern of the combination strategies: in the trial testing gefitinib plus durvalumab [26], grade 3 and 4 hypertransaminasemia were 40% and 15%, respectively, and 20% of patients had to discontinue the treatment because of TRAEs. In the phase I study testing osimertinib plus durvalumab in the second line setting [27], 38% of patients (13/34) developed interstitial lung disease (ILD), of which 15% (5 patients) were grade 3–4; treatment was discontinued in 59% of patients due to the TRAEs. The ILD frequency in the combination arm was significantly higher than in the single agent osimertinib (2.9%) or durvalumab (2%) arms. Because of the increased frequency of ILD, even the TATTON trial was ceased [27]. To date, clinical trials with a combination of TKIs and ICIs are still ongoing, most of all evaluating safety and tolerability (see Table 2 and www.clinicaltrials.gov). The reasons behind the increased toxicities are still not clearly understood, however the combinations did not demonstrate a clear synergistic anti-tumour effect, but certainly showed adverse events synergism. 

Interesting data were presented at ASCO 2019 about a phase III trial of gefitinib in combination with chemotherapy (docetaxel) versus gefitinib alone as a first-line treatment in 350 EGFR mutated patients. Although the combination has increased toxicities of grade >3 (75%, 95% C.I. 67.8–81.0 versus 49.4%, 95% C.I. 42.0–56.9; *p* < 0.001), the preliminary data seem to observe an ORR benefit (75.3%, 95% C.I. 68.3–81.1 versus 62.5%, 95% C.I. 55.1–69.3; *p* = 0.01) and increased PFS (HR 0.51, 95% C.I. 0.39–0.66; *p* < 0.001). The OS for the combination is not yet reached compared with 17 months for gefitinib alone (HR 0.45, 95% C.I. 0.31–0.65; *p* < 0.001) [59]. 

## 5. Tumour Mutational Burden (TMB) and Non-Synonymous Mutations

It is well established that cancer cells produce tumour-associated antigens (TAAs), which are processed by the APM in order to activate the effector leukocytes against them [60]. Neo-antigens can be generated by mutations, genetic rearrangements, insertions, and deletions. These tumours show greater mutational load and, consequently, may better stimulate the T cells to differentiate. The T cell clonal amplification produces a larger repertoire of anti-tumour immune cells [61]. 

Higher TMB has been related to a higher response rate and better survival outcomes in patients with NSCLC treated with ICIs [62,63,64]. The measurement of the TMB is determined by the total number of non-synonymous mutations (NSMs) per coding area of a tumour genome. NSMs result in amino acid changes in the protein sequences. The new protein epytopes (neo-antigens) are considered as foreign (“non-self”) by the host immune system and induce its activation against them. It has been hypothesized that tumours with a higher mutational burden in the presence of immune checkpoint inhibitors are more likely to induce a more robust immune response [65]. Nevertheless, it has been demonstrated that EGFR mutated patients show lower neo-antigens loads and lower numbers of NSM [66], and the latter is inversely correlated with T cell receptor β (TCRβ) clonality [67]. These data were consistent with the findings from the Cancer Genome Atlas (TCGA) program and Broad dataset analysis, where the TMB was significantly lower in EGFR mutated patients compared with the EGFR wild types. Recently, the possibility that previous chemotherapy treatments could modify the tumour mutational load has been explored. However, there seems to be no statistically significant difference in TMB from chemotherapy-naïve and chemotherapy-treated patients [68].

Nevertheless, it has recently been demonstrated that EGFR^L858R^ mutated tumours seem to have a higher TMB and a better response to ICIs [69]. In the Hastings and colleagues study, 126 patients harbouring the most common mutations in the EGFR gene (EGFR^Δ19^ and EGFR^L858R^) were treated with ICIs and were compared to 212 patients with EGFR wild types. The authors found that EGFR^Δ19^ patients showed a lower ORR compared to the EGFR wild type group (*p* = 0.002), while there was no difference between the latter group and the EGFR^L858R^. In terms of progression free survival (PFS), it was longer in the EGFR wild type patients when compared with the EGFR mutated patients, both EGFR^L858R^ and EGFR^Δ19^. Conversely, in terms of the overall survival (OS) benefit, there was no difference when comparing EGFR wild type and EGFR^L858R^, while a shorter OS was found in the EGFR^Δ19^ group (*p* = 0.069 and *p* = 0.03, respectively). These results seem to suggest that EGFR mutated tumours are not equally unresponsive to ICIs and that outcomes may vary by allele mutation. Responses to ICIs treatment have been evaluated in the EGFR resistant mutation T790M too: Haratani and colleagues [70] demonstrated that nivolumab treatment may provide worse survival outcomes in this subset of patients compared to non-mutated. Conversely, the authors suggested a possible role for ICIs in patients progressing after a first line TKI treatment who did not develop the T790M mutation: the T790M negative patients responding to ICI (nivolumab in the study) have a lower density of FOXP3+ cells, while there was no correlation between PD-L1 expression and CD8+ TILs infiltrates [70]. Although these were only preliminary results, they were consistent with Yamada and colleagues findings [71]: in this study, 27 patients with advanced NSCLC harbouring EGFR mutations (uncommon or T790M), progressing after a TKI treatment, were retrospectively analysed. The authors found better response rates and survival outcomes in patients with uncommon EGFR mutations and without T790M mutations [71]. In addition, at the ASCO 2018 meeting, data from the ImmunoTarget registry were presented: patients with T790M mutation or EGFR^Δ19^ NSCLC treated with ICIs were associated with the worst survival outcomes when compared with other mutations [72]. Taken together, these data seem to find a possible use of ICIs in some subpopulations, such as patients harbouring uncommon mutations and patients progressing after TKI without T790M mutation. Eventually, further analyses are warranted to better select EGFR mutated patients with a higher likelihood of response to ICIs.

## 6. Role of Radiotherapy

Recently, it has been of great interest to study the effects of radiotherapy (RT) on cells infiltrating the TME. It has been demonstrated that RT is able to deplete cytotoxic lymphocytes (CTLs) and natural killer (NK) cells as well as Treg [73]. Moreover, ionizing radiation can cause immunogenic cell death of cancer cells, promotes the release of tumour neo-antigens, and enhances MHC class I and PD-L1 expression [74]. In these conditions induced by radiotherapy, immunotherapy can drive a new immune infiltrate active against the tumour. However, in EGFR mutated patients, the ionizing radiations rapidly activate, in a ligand independent manner, EGFR and its downstream pathways (MAPK-ERK and PI3K/AKT), leading to the promotion of cellular proliferation and apoptosis evasion [73]. The combination of the rapid proliferation and the DNA damage repair mechanisms induced by RT leads to increased risk for errors that the tumour cells can actually tolerate in a condition of “acute” environmental change [75]. The increased errors would lead to an increased TAA release, eventually facilitating the priming of anti-tumour CTLs [76]. However, the results from the PACIFIC trial [23] do not seem to confirm this hypothesis: the EGFR mutated subgroup had an HR for PFS of 0.76, with a range of 95% C.I. crossing 1 (0.36–1.64). Although, in the durvalumab group, there were only 29 patients, and in the placebo arm, there were only 14: a cohort too small in size to reach definitive conclusions. 

## 7. Role of Angiogenesis

**Figure 2:**
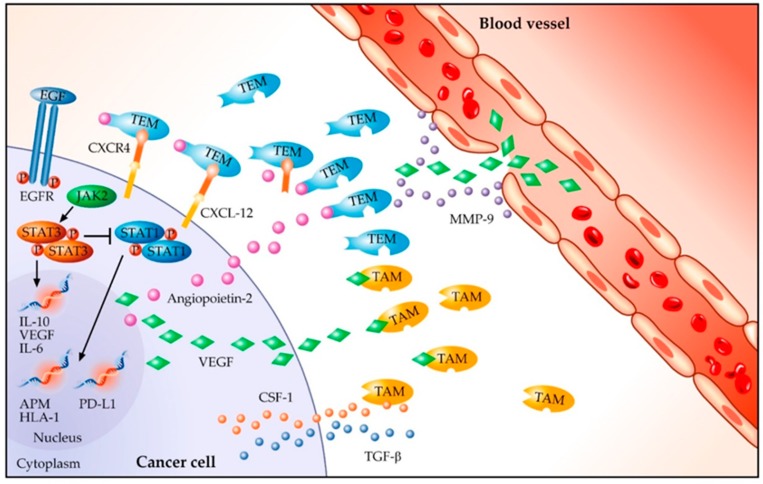
Angiogenetic factors in EGFR mutated NSCLC. EGF, Epidermal Growth Factor; EGFR, Epidermal Growth Factor Receptor; STAT1/3, Signal Transducer and Activator of the Transcription1/3; JAK2, Janus Activated Kinase 2; IL, Interleukin; VEGF, Vascular Endothelial Growth Factor; APM, Antigen Presenting Machine; HLA-1, Human Leukocyte Antigen-1; PD-L1, Programmed cell Death Ligand 1; CSF-1, Colony Stimulating Factor-1; TAM, Tumour Associated Macrophage; TEM, TIE-2 expressing monocytes; CXCR4, C-X-C chemokine receptor type 4; CXCL12, C-X-C motif chemokine 12; TGFβ, transforming growth factor β; MMP-9, matrix metalloproteinases 9.

There are several preclinical data supporting the role of angiogenesis inhibitors in modulating, directly or indirectly, the anti-tumour immune response [77]. Indeed, the neo-angiogenesis in the TME, induced by the tumour cells, produces abnormal vessels, thus promoting a hypoxic and acid milieu. These factors contribute to the immunosuppressive effect through the activation of Tregs and the inhibition of the effectors’ T cell functions [77,78]. Moreover, VEGF and bFGF (basic fibroblast growth factor) have been related to a downregulation of leukocytes adhesion in nude mice models [79], resulting in low trafficking of immune cells in TME [77]. In addition, it has been demonstrated that tumour cells are able to produce angiopoietin-2, which recruits TIE-2 expressing monocytes (TEM) into the tumour site. The hypoxia of certain areas of the tumour stimulates TAMs to produce VEGF-A, thus stimulating chemotaxis of endothelial cells and macrophages. In addition, TAMs produce matrix metalloproteinases 9 (MMP9) that are responsible for the degradation of the extracellular matrix (ECM), releasing even more VEGF-A [80]. In conclusion, a positive feedback is established between tumour cells, TEMs, and TAMs: tumour cells recruit TEMs by expressing CXCL12, receptor to CXCR4, which is located on the TEM surface, and TAMs through CSF-1 and TGFβ [80] (Figure 2). 

As further confirmation, in CSF-1 null mice, TAM infiltration was lower and the vasculature network showed impairment, which is definitive proof that TAM infiltrate is able to activate the angiogenesis switch [81]. Taken together, the abovementioned preclinical data suggest that the EGFR mutated population could benefit from treatment with angiogenesis inhibitors and ICIs in order to modulate the TME. 

Indeed, clinical data from the imPOWER150 phase III trial [82] are already available: this trial, which allowed the enrolment of oncogene-addicted patients (including EGFR mutated, any line of treatment), compared bevacizumab, a monoclonal antibody directed against VEGF, carboplatin, and paclitaxel (BCP) versus atezolizumab plus BCP (ABCP). In the EGFR mutated subgroup, this trial demonstrated improvements both in PFS (10.2 months versus 6.9 months, HR 0.61, 95% C.I. 0.36–1.03) and OS (not estimable versus 18.7 months, HR 0.61, 95% C.I. 0.29–1.28) when comparing ABCP and BCP. The results were even more interesting in the subgroup of sensitizing EGFR mutation (EGFRΔ19 and EGFRL858R), where the HR for PFS was 0.41 (95% C.I. 0.23–0.75) and HR for OS was 0.31 (95% C.I. 0.11–0.83). Further, 50 patients (63% of the total EGFR mutated population) had previously received TKI therapy: in this subgroup, the median PFS was 9.7 months in the ABCP treatment group versus 6.1 months (BCP arm), with HR for PFS 0.42 (95% C.I. 0.22–0.80); while the median OS was not estimable (NE) in the ABCP group versus 17.5 months in the BCP treatment arm (HR OS 0.39, 95% C.I. 0.14–1.07). Despite some interesting HR point estimates, often the upper bound 95% C.I. crossed 1: this was probably due to the small sample size. However, these results support a role for immunotherapy if it is associated with antiangiogenics molecules: indeed, the survival benefits were not as brilliant in the third arm of treatment of this study using only immunotherapy plus chemotherapy (ACP). Further clinical trials are ongoing to evaluate the safety, feasibility, and activity of a combination of ICIs with antiangiogenics in EGFR mutated populations of NSCLC (www.clinicaltrials.gov).

## 8. Conclusions

In the immunotherapy era, whereas the NSCLC population gained great survival benefits, patients with EGFR addicted diseases are to be considered orphans. The reasons for these unsatisfying results needs to be investigated in the characteristics of the TME and in the dynamic relation between tumour cells and immune infiltrate. Right now, a biomarker-driven selection, through the evaluation of PD-L1 or TMB, of the subgroup of EGFR mutated patients benefitting from ICIs is far from reality. However, in order to overcome this “primary resistance” to the immunotherapy treatment in such patients, a tool that is able to interact and modify an immune–excluded TME is needed. Even though there were interesting preclinical data, RT treatment did not produce concrete results in the clinical trials. Angiogenesis inhibitors seem to be able to provide the changes needed and their combination with ICIs could, eventually, re-integrate immunotherapy in the treatment strategy of EGFR mutated NSCLC patients. However, further data and clinical trials are needed to confirm this hypothesis. 

## Figures and Tables

**Table 1:** Studies of ICIs where patients with EGFR mutation were included.

Trial	Phase	Treatment Arms	Line of Treatment	Number of Patients Mutated Versus Wild Type	Results Mutated Versus Wild Type (95% C.I. Range)	Reference
*CheckMate 057*	III	Docetaxel vs. Nivolumab	≥2	82 versus 340	HR OS 1.18 (0.69–2.00) HR PFS 1.45 (0.90–2.37) versus HR OS 0.66 (0.51–0.86) HR PFS 0.83 (0.65–1.06)	[15]
*KEYNOTE-010*	II-III	Docetaxel vs. Pembrolizumab	≥2	86 versus 875	HR OS 0.88 (0.45–1.70) HR PFS 1.79 (0.94–3.42) versus HR OS 0.66 (0.55–0.80) HR PFS 0.83 (0.73–0.98)	[16]
*POPLAR*	II	Docetaxel vs. Atezolizumab	≥2	19 versus 147	HR OS 0.99 (0.29–3.40) HR PFS N/A versus HR OS 0.73 (0.53–0.99) HR PFS N/A	[17]
*OAK*	III	Docetaxel vs. Atezolizumab	≥2	85 versus 628	HR OS 1.24 (0.71–2.18) HR PFS N/A versus HR OS 0.69 (0.57–0.83) HR PFS N/A	[18]
*ATLANTIC trial (cohort 1)*	II	Durvalumab	≥3	97	12% ORR (PD-L1 ≥25%) 4% (PD-L1 <25%) **	[48]
*NCT02879994*	II	Pembrolizumab	1	11 *	No Responses **	[24]

* The study was ceased because of lack of efficacy. HR, Hazard Ratio; OS, overall survival; PFS, progression free survival; N/A not available; ORR Objective Response Rate; PD-L1, programmed cell death ligand-1; ICI, immune checkpoint inhibitors. ** In this cohort, only mutated patients were enrolled.

**Table 2:** Studies of ICIs combined with TKIs.

TKI Treatment	ICI Treatment	Inclusion Criteria	Number of Patients	ORR	Grade 3–4 TRAEs	Discontinuation Rate Due to TRAEs	Status	Reference
*Erlotinib*	Nivolumab	EGFR +, TKI treated, or naive	21	15%	10% diarrhoea	N/A	Completed	[46]
*Erlotinib*	Atezolizumab	EGFR+, TKI naïve, or previously treated but not with TKI	28	75%	7% hypertransa-minasemia, 7% rash, 7% fever	N/A	Active, Not Recruiting	[47]
*Gefitinib*	Durvalumab	EGFR+, TKI naive	20	N/A *	55% hepatic	20%	Active, Not Recruiting	[48]
*Osimertinib*	Durvalumab	EGFR +, TKI treated, or naive	34	N/A *	15% ILD	59%	Active, Not Recruiting	[20]

ORR, Overall Response Rate; N/A, Not Available; TKI, Tyrosine Kinase Inhibitor; ILD, Interstitial Lung Disease; TRAEs, treatment-related AEs. * ongoing trials.
